# Navigating Oral Cancer Management: Combining Artificial Intelligence and Clinical Guidelines for Optimal Decision-Making

**DOI:** 10.1055/a-2852-9642

**Published:** 2026-08-14

**Authors:** Mario Augusto Ferrari de Castro, Rogério Aparecido Dedivitis, Leandro Luongo de Matos, Paulo Vitor Sóla Gimenes, Bruno Pelinson Fogaça Duarte, Luiz Paulo Kowalski

**Affiliations:** 1 Metropolitan University of Santos, Santos, Brazil; 2 Department of Head and Neck Surgery, Hospital das Clínicas, University of São Paulo School of Medicine, São Paulo, Brazil; 3 Department of Head and Neck Surgery, Instituto do Câncer do Estado de São Paulo, Hospital das Clínicas, Faculdade de Medicina, Universidade de São Paulo (ICESP HCFMUSP), São Paulo, Brazil; 4 Irmandade da Santa Casa de Misericórdia de São Carlos, São Carlos, Brazil; 5 Metropolitan University of Santos, Santos, Brazil; 6 Department of Head and Neck Surgery, University of São Paulo School of Medicine, São Paulo, Brazil

**Keywords:** artificial intelligence, clinical practice guideline, oral cancer, carcinoma, squamous cell

## Abstract

**Introduction:**

Decision-making is one of the most difficult and important tasks performed by surgeons. Clinical guidelines and, more recently, Artificial Intelligence (AI) have been implemented to support these decisions. However, there is a paucity of research exploring the impact of AI on surgical decision-making strategies.

**Objective:**

To evaluate the accuracy of AI in informing clinical decision-making for the treatment of advanced oral cancer.

**Methods:**

Structured questions based on seven selected clinical recommendations were formulated and processed through multiple large language model (LLM) platforms. Claude (Sonnet 3.5) was used to identify discrepancies between established guidelines and LLM-generated responses. Subsequently, three subject matter experts assessed the identified differences to evaluate their clinical significance.

**Results:**

Analysis of the 28 generated responses revealed that 21 (75%) showed congruence between LLM outputs and established guideline recommendations. In three instances, guidelines provided significantly more comprehensive content than LLM responses. One LLM response contained additional relevant information, while three responses were contradictory to the guidelines.

**Conclusion:**

The LLMs demonstrated 75% concordance with guideline recommendations and should be considered complementary tools for established clinical guidelines.

## Introduction

Traditional clinical decision support tools have the potential to reduce variability and mitigate risks; however, their clinical adoption is often hindered by suboptimal accuracy and time-consuming requirements of manual data acquisition and entry. Artificial intelligence (AI) generally refers to computer systems that can learn from raw data, utilizing approaches such as machine learning, deep learning, and reinforcement learning. In contrast to conventional clinical decision-support systems that rely on predefined rules to generate codes and algorithms, AI models learn from examples. 1

Management of oral cavity squamous cell carcinoma is fundamentally surgery-based, yet key therapeutic decisions remain controversial. Clinically N0 neck management still varies between observation, elective neck dissection, and sentinel node biopsy, reflecting ongoing debate on oncologic equivalence and morbidity. 2 3 Depth of invasion thresholds used to trigger neck treatment (and their site specificity) remain disputed. 4 Adjuvant indications are also contested in “intermediate-risk” settings, including pT4aN0 disease upstaged by mandibular invasion and pN1 disease without extranodal extension, where the survival benefit of PORT is inconsistently demonstrated. 5 Increased lymph node density – defined as the ratio of positive lymph nodes to total nodes excised – is independently associated with poorer overall survival and can refine prognostic stratification beyond conventional nodal staging. 6 Finally, perioperative immunotherapy is rapidly evolving – early neoadjuvant regimens appear feasible, but optimal selection, sequencing, and long-term benefit in resectable disease are still being defined. 7 Given these uncertainties, adherence to current evidence-based guidelines is essential to standardize care and responsibly navigate controversies. 4

In the context of oral cancer, numerous guidelines exist, each characterized by varying development methods, quality, and strength of evidence. 8 For instance, the Latin American guidelines for the care of patients with head and neck cancer were developed by a panel of 41 head and neck cancer experts who systematically followed a modified Delphi process. This consensus document encompasses 12 sections, with Section 4 specifically addressing the evidence-based management of patients with squamous cell carcinoma of the oral cavity or oropharynx, encapsulating recommendations from 10 to 16. 9

Platforms such as ChatGPT, Claude, Gemini, and Perplexity are grounded in a diverse array of texts sourced from the internet, including books, articles, and websites. These platforms can generate comprehensive, fluent and multilingual responses across various fields, including medicine. 10 However, few studies have investigated the capacity of large language models to assist healthcare professionals in interpreting clinical guidelines.

Demonstrating the value and efficiency of healthcare AI tools is essential, yet insufficient for driving their adoption and scaling within clinical practice. Even where data are abundant, important implementation challenges persist; the most critical which may involve fostering trust among both the public and healthcare providers in AI-based solutions. 11

The aim of the present study was to evaluate the accuracy of AI in facilitating clinical decision-making for the treatment of oral cancer in comparison with established clinical guidelines.

## Methods

The Latin American Consensus on the Treatment of Head and Neck Cancer comprises 12 sections, including Head and Neck Cancer Staging, Histopathologic Evaluation, Surgery and Medical Oncology of Oral Cavity, Oropharynx, Larynx and Hypopharynx, Management of Recurrent and Metastatic Disease, Reconstruction, Rehabilitation and Radiation therapy. Within this framework, 48 recommendations were established for patient care, considering resource availability and oncologic benefits. 9

The present study focuses on Section 3, which addresses head and neck surgery for the oral cavity, presenting evidence for various neck approaches (Recommendations 10 to 12), and Section 4, which discusses clinical oncology for the oral cavity, providing evidence for the systemic treatment of locally advanced cases (Recommendations 13 to 16). 9

The methodological approach involved developing targeted inquiries corresponding to each of the seven recommendations. A panel of three subject matter experts collaborated to formulate these questions through a consensus-based process, ensuring comprehensive coverage of critical components within each guideline recommendation. The interrogative framework was systematically designed with a tripartite structure: an introductory segment establishing the target persona, a specific clinical task description, and explicit parameters regarding the desired response format.

The standardized prompt used across all platforms was as follows: “Assume the role of an experienced head and neck surgeon with comprehensive expertise in evidence-based medicine. Respond to the clinical inquiries regarding oral cavity carcinoma management and treatment protocols presented below. Your answers must exclusively derive from peer-reviewed scientific literature. Each response should be concise, contained within a single paragraph, and reflect the most current and pertinent evidence available.” The large language models (LLMs) evaluated were ChatGPT-4 (OpenAI, Inc., San Francisco, CA, USA), Claude (Anthropic, PBC, San Francisco, CA, USA), Gemini (Google LLC, Mountain View, CA, USA), and Perplexity (Perplexity AI, Inc., San Francisco, CA, USA). The most recent paid versions available at the time of study development were used. We selected the platforms for analysis based on their widespread adoption. The Copilot platform was excluded as it shared the same database as ChatGPT during the study period. Therefore, the researchers had no conflicts of interest influencing this selection. The models interpret data from multiple languages.

ChatGPT is a language model developed by OpenAI based on GPT architecture, trained on extensive textual data to understand and generate contextually relevant responses. It processes queries, interprets intentions, and produces human-like text, with applications in education, customer service, content creation, and research support. 12

Claude is an advanced LLM that utilizes transformer architecture and neural attention mechanisms. It incorporates Constitutional AI to ensure ethical adherence in its responses, generating content based on context and learned patterns. 13

Gemini, introduced by Google in December 2023, represents a new generation of LLMs with multimodal processing capabilities for text, code, and images. It leverages attention mechanisms to prioritize various informational components and showcases significant advancements AI applications. 14

Perplexity, established in August 2022, functions as an AI-driven search and conversation engine. It employs advanced language models, including GPT-4 and Claude 3, to process natural language queries and provide contextualized responses, differentiating itself from traditional search engines by synthesizing information from multiple sources with direct citations. 15

The panel of three head and neck surgery specialists engaged in a collaborative evaluation process to refine the LLM-generated responses through consensus methodology. This refinement involved systematically eliminating non-essential elements, such as bibliographic references and verbosity, thereby enhancing the precision and recommendation-centric focus of the content. This curation process ensured that the resulting material adhered strictly to relevant clinical guidelines while optimizing clarity and utility.

The methodological framework for evaluation encompassed the systematic acquisition and analytical examination of responses generated by each language model. Claude (Sonnet 3.5) was used as the reference standard for identifying discrepancies between guideline recommendations and LLM-generated content, based on its demonstrated text generation capabilities. 13 To optimize comparative analysis, a tripartite presentation structure was implemented: initially presenting the clinical query, followed by the corresponding guideline recommendation, and concluding with the LLM-generated response.

The standardized prompt utilized for comparative analysis was: “I am conducting a systematic evaluation of congruence between oral cancer guideline recommendations and LLM platform-generated responses to specific clinical inquiries. I will provide the question, guideline recommendation, and corresponding LLM response for comparative assessment. Please objectively delineate, within a single paragraph, the principal substantive discrepancies between the guideline recommendations and the platform's response, focusing exclusively to the central elements of the clinical question.”

This standardization protocol ensured consistent evaluation parameters across all platforms while maintaining precise focus on essential clinical guidance components. The LLM-generated responses underwent a systematic refinement process in which the panel of three head and neck surgery specialists used consensus methodology to eliminate non-essential content, further enhancing the recommendation-centric nature of the AI-generated material.

## Results

Table 1 presents the guideline recommendations, the corresponding formulated inquiries, and the responses generated by each LLM platform. Accompanying each response, the substantive deviations from the original recommendations, as identified through systematic analysis by the Claude platform, are outlined.

**Table 1:** The Guideline Recommendations, the Corresponding Formulated Inquiries, and the Responses Generated by Each LLM Platform

Recommendations regarding the management of patients with oral cavity or oropharynx squamous cell carcinoma	Chat GPT		Claude		Gemini		Perplexity	
	Answer	Difference	Answer	Difference	Answer	Difference	Answer	Difference
10. Sentinel lymph node biopsy (SLNB) should be employed in patients with stages I and II oral SCC when all the resources, such as appropriate preoperative imaging, specialized head and neck cancer radiologist, nuclear medicine services, radiolabeled markers, an adequate pathologist, and immunohistochemistry techniques, are available. Otherwise, SND should continue to be the standard of care for those patients. Active surveillance of the neck should not be recommended. “Regarding the diagnosis and surveillance of the neck in patients with stages I and II oral SCC, when should sentinel lymph node biopsy be employed? If it is not feasible, what should be the standard care in this context? Is active surveillance of the neck recommended?”	SLNB is indicated in clinically node-negative (cN0) necks for patients with stages I and II oral SCC to detect occult metastasis. It is particularly valuable in centers with expertise, as it provides accurate staging while minimizing morbidity compared to elective neck dissection (END). SLNB is most effective when combined with reliable imaging and intraoperative mapping techniques. If SLNB is not available or feasible, END is the standard of care. Active surveillance is not generally recommended due to the poor outcomes associated with delayed intervention when nodal disease becomes clinically apparent. Surveillance may only be considered in exceptional cases where surgical risks outweigh potential benefits and should involve rigorous follow-up protocols.	The core differences between the guideline and ChatGPT responses are: 1) The guideline is more specific and detailed about the required resources for SLNB implementation, while ChatGPT provides a more general recommendation about "centers with expertise"; 2) ChatGPT elaborates on active surveillance by mentioning specific scenarios where it might be considered and emphasizes the need for rigorous follow-up protocols, whereas the guideline makes a clear, absolute statement against active surveillance without exceptions.	SLNB should be considered for T1-T2N0 oral SCC with depth of invasion >4mm when expertise and infrastructure are available, as it provides staging information with less morbidity than END. When SLNB is not feasible due to technical or resource limitations, END remains the standard of care for early-stage oral SCC with depth of invasion >4mm. Active surveillance alone (regular clinical examination and imaging) should only be considered for very early tumors with depth of invasion <4mm or in patients unfit for surgery.	The core differences between the guideline and Claude's response are: 1) The guideline makes no specific mention of tumor depth of invasion as a criterion for SLNB, while Claude explicitly recommends SLNB for tumors >4mm depth; 2) The guideline provides a more detailed list of specific institutional requirements for SLNB implementation, while Claude mentions these more generally; and 3) The guideline categorically states that active surveillance should not be recommended, while Claude allows for active surveillance in select cases (very early tumors <4mm depth or surgically unfit patients).	SLNB is increasingly being considered a viable alternative to END in this patient population. However, SLNB requires meticulous technique and experienced surgeons to ensure accuracy. If SLNB is not feasible due to lack of expertise or resources, the standard of care remains END of levels I-III. Active surveillance of the neck, with close clinical follow-up and imaging studies, is another option, particularly for patients with very low-risk tumors (e.g., small, well-differentiated T1 lesions) who may not benefit from immediate surgery.	While the guideline explicitly lists specific requirements for SLNB implementation, Gemini provides a more general statement about "meticulous technique and experienced surgeons." Most significantly, there is a direct contradiction regarding active surveillance - the guideline explicitly states it "should not be recommended," while Gemini suggests it as a viable option for low-risk tumors.	SLNB is an effective diagnostic tool for staging the cN0 neck in early OSCC when available. If not feasible, END remains the standard of care for most cases. Active surveillance alone is generally not recommended except in very select low-risk scenarios.	The core differences between the guideline and LLM responses are: 1) The guideline provides specific requirements for SLNB implementation, while the LLM response lacks these detailed technical prerequisites; 2) The guideline makes an absolute statement against active surveillance, while the LLM leaves room for surveillance in "very select low-risk scenarios," which represents a significant divergence in clinical approach.
11. A lymph node yield (LNY) higher than 18 is a quality metric in head and neck surgery, specifically in patients undergoing elective neck dissection. The surgical technique can significantly impact the LNY, but also it is possible to improve this evaluation with the application of more rigorous specimen processing by the pathology team. Subsequently, this measure could yield more accurate nodal staging and ultimately drive more appropriate use of adjuvant therapy. “What is the minimum number of lymph nodes dissected (LNY) in elective lymph node dissection that can be considered adequate to provide more accurate nodal staging and ultimately lead to the more appropriate use of adjuvant therapy?”	In elective lymph node dissection (END) for oral squamous cell carcinoma (SCC), dissecting a minimum of 18 lymph nodes is widely accepted as adequate for accurate nodal staging.	Both agree on the minimum threshold of 18 lymph nodes.	According to large-scale studies focusing specifically on END in oral SCC, a minimum LNY of 18 nodes should be achieved for selective neck dissection to ensure adequate staging accuracy.	The core differences between the guideline and Claude's response are minimal in terms of the recommended numerical threshold (both specify 18 nodes)	A yield of at least 18 lymph nodes was associated with a significantly higher rate of identifying positive nodes compared to dissections with fewer than 18 nodes.	The core differences between the guideline and Gemini's response lie in their depth and scope.	Based on the available evidence, the minimum number of lymph nodes that should be dissected in an END to be considered adequate for accurate nodal staging is 18.	Both responses agree on the minimum threshold of 18 lymph nodes
12. Selective neck dissection (SND) is an acceptable alternative to CND or RND in patients with stage T1 or T2 and cN1 oral cavity SCC and for very selected cN2 cases, sited at levels I or II. However, radical neck dissection (RND) and comprehensive neck dissection (CND) are still the standard of care in patients with oral cancer with advanced T-stage disease (T3 or T4) or cN2 and cN3 disease, which require primary surgical treatment. “What is the surgical treatment of the neck in patients with stage T1 or T2 and cN1 oral cavity SCC or in very selected cases of cN2 located in levels I or II? What is the standard surgical treatment of the neck in patients with oral cancer with advanced T-stage disease (T3 or T4) or cN2 and cN3 disease requiring primary surgical treatment?”	In patients with T1 or T2 oral cavity SCC and cN1 disease, or very selected cases of cN2 (limited to levels I or II), a SND is the recommended surgical approach. or patients with advanced T-stage (T3 or T4) or cN2/cN3 disease requiring primary surgical treatment, the standard of care is a CND or modified radical neck dissection (MRND).	both recommend SND for early-stage disease (T1-T2/cN1) and selected cN2 cases in levels I/II. However, for advanced disease (T3-T4 or cN2-N3), the guideline specifically maintains RND alongside CND as standard options, while the LLM response only mentions CND/MRND, omitting RND as a standard treatment option.	In patients with T1-T2/cN1 or selected cN2 (levels I/II) oral SCC, current evidence supports SND. For patients with T3-T4 disease or cN2-N3 disease, comprehensive MRND (levels I-V) remains the standard of care.	The core differences between the guideline and Claude's response lie in the specification of neck dissection types and their presentation. The guideline presents SND as an "acceptable alternative" to CND/RND for early stages, while Claude more definitively "supports" SND as the primary approach. For advanced stages, the guideline specifically mentions both RND and CND as standard of care, while Claude only specifies MRND (levels I-V).	For early-stage oral SCC (T1-T2, cN1 or select cN2 in levels I-II), a supraomohyoid neck dissection (SND) is often considered adequate. In contrast, for advanced T-stage (T3-T4) or more extensive nodal disease (cN2-cN3), a CND, including levels I-V, is generally recommended.	Both agree on selective approaches for early-stage disease and comprehensive treatment for advanced cases	For patients with T1-T2 tumors and cN1 disease or selected cases of cN2 disease limited to levels I or II: SND of levels I-III is generally recommended. In some cases, extended SND to include level IV may be considered, especially if there is clinical suspicion of involvement. For patients with advanced T-stage disease (T3 or T4) or cN2-N3 disease requiring primary surgical treatment: CND is recommended, typically including levels I-IV	The key differences between the guideline and Perplexity's response are: 1) The guideline specifically mentions RND and CND as standard of care for advanced disease (T3-T4, cN2-N3), while Perplexity's response is less specific, only mentioning "comprehensive neck dissection" without detailing the type; 2) Perplexity specifies levels I-III for SND and suggests possible extension to level IV, while the guideline doesn't specify the exact levels to be dissected; 3) Perhaps most importantly, Perplexity's response is incomplete as it only recommends levels I-IV for advanced disease, while standard CND should include level V as well, which is correctly implied in the guideline's recommendation of RND/CND.
13. There is a strong recommendation for adjuvant concurrent chemoradiation over adjuvant radiation therapy alone primarily for patients with positive surgical margins and/or metastatic lymph nodes with ENE. The presence of more than two positive lymph nodes is also an indication for postoperative chemoradiation. The presence of perineural invasion (PNI), perivascular invasion, involvement of multiple lymph nodes, and lymph node involvement at low cervical levels (IV and V) could, in highly selected cases and after multidisciplinary discussion, be considered for concurrent chemoradiation. “In which cases is adjuvant concurrent chemoradiation recommended instead of adjuvant radiation therapy alone?”	Adjuvant concurrent chemoradiation is recommended over radiation therapy alone in cases with high-risk pathological features following surgical treatment of head and neck squamous cell carcinoma, including oral cavity squamous cell carcinoma. These high-risk features include: Extranodal extension (ENE): The presence of tumor extension beyond the lymph node capsule is a significant indicator for chemoradiation, as it is associated with poorer locoregional control and survival. Positive surgical margins: If tumor cells are identified at the surgical margin, chemoradiation offers improved outcomes by addressing residual microscopic disease.	The guideline explicitly includes having more than two positive lymph nodes as a strong indication for chemoradiation, which is absent in the LLM response. Additionally, the guideline provides more nuanced recommendations by specifying secondary considerations that could warrant chemoradiation after multidisciplinary discussion - these details are completely omitted in the LLM response. While both correctly identify positive margins and ENE as primary indications, the LLM response is notably more limited in scope and misses several key clinical scenarios outlined in the guideline.	Adjuvant concurrent chemoradiation (CCRT) is recommended over radiation alone in cases with high-risk pathological features, specifically: ENE and/or positive surgical margins (R1/R2).	While both agree on positive margins and ENE as primary indications, the guideline is more comprehensive and includes additional specific indications that Claude omits: 1) The guideline explicitly states that >2 positive lymph nodes is an indication for chemoradiation, which Claude doesn't mention, 2) The guideline provides additional considerations for chemoradiation in selected cases after multidisciplinary discussion, whereas Claude's response excludes these situations entirely.	Here are some scenarios where the evidence supports the use of adjuvant concurrent chemoradiation over radiation therapy alone: Positive surgical margins: If the surgical resection has positive margins, indicating microscopic residual disease, the addition of chemotherapy to radiation therapy improves local control and survival. Extracapsular spread (ECS): ECS in lymph node metastases is a strong predictor of recurrence. Concurrent chemoradiation is recommended to address the increased risk of both local and distant disease. The presence of multiple involved lymph nodes suggests a more aggressive tumor biology. Concurrent chemoradiation offers a more intensive treatment approach to improve outcomes. PNI indicates the tumor's ability to spread along nerves, increasing the risk of local recurrence. Concurrent chemoradiation is often recommended to enhance local control.	The core differences between the guideline and Gemini's response are in their level of specificity and hierarchy of indications. The guideline clearly establishes a strong primary tier of indications (positive margins and ENE) and explicitly states that more than two positive nodes is an independent indication, while other factors require multidisciplinary discussion. In contrast, Gemini's response presents all indications (margins, ECS, multiple nodes, PNI) as equally weighted recommendations without this hierarchical distinction and doesn't specify the threshold for "multiple positive nodes" nor mention the need for multidisciplinary discussion for secondary risk factors. Additionally, Gemini lists perineural invasion as a standalone indication, while the guideline groups it with other risk factors that require careful case selection and team discussion.	Adjuvant concurrent chemoradiation is recommended instead of adjuvant radiation therapy alone in the following cases for patients with oral cavity SCC: High-Risk Pathological Features Positive surgical margins ENE pT3 or pT4 tumors N2 or N3 nodal disease Lymph node metastasis at levels IV or V PNI Lymphovascular invasion Other factors Higher nodal disease burden (two or more positive lymph nodes) Patients with unresectable disease when surgery is not feasible	The guideline provides a clear hierarchical recommendation, strongly indicating chemoradiation only for positive margins and ENE, with >2 positive nodes as a separate indication, and other factors requiring multidisciplinary discussion. In contrast, Perplexity's response lists all risk factors (including pT3/T4 status and unresectable disease) as equal indications without distinguishing between strong and conditional recommendations and doesn't emphasize the need for multidisciplinary discussion for secondary risk factors. Additionally, Perplexity incorrectly includes unresectable disease in adjuvant therapy indications, which is a fundamentally different clinical scenario requiring primary rather than adjuvant treatment.
14. Adjuvant (postoperative) concurrent chemoradiation with once a week cisplatin (40 mg/m ^2^ ) can be used instead of the high-dose regimen (100 mg/m ^2^ ) once every 3 weeks. “What regimen can be used instead of the high-dose regimen (100 mg/m ^2^ ) every 3 weeks for adjuvant (postoperative) concurrent chemoradiation with cisplatin?”	For adjuvant concurrent chemoradiation with cisplatin, an alternative to the high-dose regimen of 100 mg/m ^2^ every 3 weeks is the low-dose weekly cisplatin regimen of 40 mg/ m ^2^ given once per week.	There are no significant differences between the guideline and the LLM response.	According to recent clinical trials and meta-analyses, weekly cisplatin at 40 mg/m ^2^ can be considered an acceptable alternative to the high-dose regimen, particularly in patients at risk for toxicity.	The core similarity between both responses is the recommendation of weekly cisplatin at 40 mg/m ^2^ as an alternative to the high-dose regimen.	A widely studied alternative is weekly cisplatin at a lower dose (typically 30-40 mg/m ^2^ ).	While the guideline provides a precise dosing recommendation of 40 mg/m ^2^ weekly cisplatin, Gemini's response is less specific, presenting a range of 30-40 mg/m ^2^ and lacks explicit identification of this as a postoperative concurrent chemoradiation regimen.	Several alternative regimens can be used instead of the high-dose cisplatin (100 mg/m ^2^ ) every 3 weeks for adjuvant concurrent chemoradiation: Weekly low-dose cisplatin 30-40 mg/m ^2^ weekly during radiotherapy daily low-dose cisplatin 20 mg/m ^2^ daily during radiotherapy.	The guideline provides a single, specific alternative regimen (40 mg/m ^2^ weekly cisplatin), while the LLM response suggests multiple options including both weekly (30-40 mg/m ^2^ ) and daily (20 mg/m ^2^ ) regimens.
15. Induction (neoadjuvant) chemotherapy may be considered in unresectable or borderline resectable oral cavity tumors in highly selected cases with multidisciplinary input. “What is the option for patients with unresectable or borderline resectable oral cavity tumors in highly selected cases with multidisciplinary input?”	For patients with unresectable or borderline resectable oral cavity tumors, an option in highly selected cases, with input from a multidisciplinary team, is induction chemotherapy followed by reassessment for surgical resectability or definitive chemoradiation therapy.	Both responses align on the use of induction chemotherapy for unresectable/borderline resectable cases and emphasize multidisciplinary input	According to recent clinical evidence, induction chemotherapy (IC) with TPF (docetaxel, cisplatin, and 5-fluorouracil) followed by local therapy can be considered for highly selected cases of unresectable or borderline resectable oral cavity tumors.	Both responses align on the core recommendation of induction chemotherapy for unresectable or borderline resectable oral cavity tumors in selected cases,	In these highly selected cases of unresectable or borderline resectable oral cavity tumors, neoadjuvant chemotherapy (NACT) followed by reassessment is a valuable option.	While both mention induction/neoadjuvant chemotherapy as an option, the guideline is more cautious in its recommendation, using "may be considered" and emphasizing multidisciplinary input, whereas Gemini's response is more definitive in stating it is "a valuable option" and specifically mentions the need for reassessment after chemotherapy.	Induction (neoadjuvant) chemotherapy may be considered as an option for patients with unresectable or borderline resectable oral cavity tumors in highly selected cases with multidisciplinary input.	There are no significant core differences between the guideline and the LLM response.
16. There is no evidence to prescribe cetuximab concurrent with radiation therapy in patients with oral cavity unsuitable to receive cisplatin. “Is there scientific evidence to prescribe cetuximab concurrent with radiation therapy for patients with oral cavity tumors who are not suitable to receive cisplatin?”	Yes, there is scientific evidence supporting the use of cetuximab concurrent with radiation therapy for patients with oral cavity (SCC) who are not suitable candidates for cisplatin	The core difference lies in their contradictory positions - while the guideline categorically states there is no evidence supporting cetuximab with radiation therapy for cisplatin-unsuitable oral cavity cancer patients, ChatGPT incorrectly suggests there is scientific evidence supporting this approach.	Based on current evidence, the use of cetuximab with radiation therapy specifically for oral cavity squamous cell carcinoma lacks robust supportive data.	The core substance of both responses is identical - both clearly state there is no strong evidence supporting cetuximab with radiation therapy for oral cavity cancer in cisplatin-unsuitable patients.	The evidence for cetuximab concurrent with radiation therapy in oral cavity tumors specifically is somewhat limited compared to its use in other head and neck subsites like oropharynx. However, some studies suggest it can be a viable option.	The core difference lies in the stance on cetuximab use - while the guideline takes a definitive position stating there is "no evidence" to support cetuximab with radiation therapy in cisplatin-unsuitable oral cavity cancer patients, Gemini's response is more permissive, suggesting it could be "a viable option" despite limited evidence, which potentially could lead to inappropriate treatment choices.	Yes, there is scientific evidence supporting the use of cetuximab concurrent with radiation therapy for patients with oral cavity tumors who are not suitable to receive cisplatin.	The core difference between the recommendations is fundamentally contradictory - while the guideline categorically states there is NO evidence to support cetuximab use with radiation therapy in cisplatin-unsuitable oral cavity cancer patients, Perplexity incorrectly asserts that there IS scientific evidence supporting this approach.

Assume the role of an experienced head and neck surgeon with extensive expertise in evidence-based medicine. Respond to the clinical questions regarding the management and treatment of oral carcinoma provided below. Your answers must be STRICTLY based on information from articles published in peer-reviewed scientific journals. Each response should be concise, limited to a single paragraph, and summarize the most recent and relevant evidence.

Analysis of the 28 generated responses revealed that 21 (75%) exhibited substantial congruence between LLM-generated recommendations and established guidelines, as confirmed through specialist consensus evaluation. Three instances showed significantly more comprehensive content within the guidelines compared with LLM outputs. Additionally, one response contained supplementary relevant information not included in the corresponding guidelines, whereas three responses presented direct contradictions to the guideline recommendations ( Table 2 ). Table 3 delineates these findings stratified by individual LLM platform. ChatGPT, Gemini, and Perplexity each presented one discordant answer, in different questions. Claude provided further information than the guidelines in one question, whereas the guidelines provided more comprehensive information than ChatGPT, Claude, and Perplexity in one question each.

**Table 2:** Analysis of the 28 Generated Responses

Question	Chat GPT	Claude	Gemini	Perplexity
10	similar	platform +	discordant	similar
11	similar	similar	similar	similar
12	similar	similar	similar	guideline +
13	guideline +	guideline +	similar	similar
14	similar	similar	similar	similar
15	similar	similar	similar	similar
16	discordant	similar	similar	discordant

+: with relevant additional information.

**Table 3:** Findings Stratified by Individual LLM Platform

	Chat GPT	Claude	Gemini	Perplexity
Similar content	5	5	6	5
Guideline with further content	1	1	0	1
Platform with further content	1	1	0	0
Discordant content	0	0	1	1

## Discussion

This investigation examined the potential role of AI in relation to medical guidelines. Medical guidelines serve as a mechanism to preserve physician autonomy and clinical decision-making flexibility, offering evidence-based recommendations suitable for most patients without imposing mandatory protocols for all clinical scenarios. 16 Over time, these guidelines have increasingly been used as supporting evidence in legal proceedings. In head and neck oncology, rigorous evidence-based clinical practice guidelines standardize evaluation and treatment algorithms, enhancing multidisciplinary decision-making and reducing variability in complex therapeutic choices across diverse clinical scenarios. 17 Table 1 presents the guideline recommendations, the corresponding formulated inquiries, the responses elicited from each LLM, and the substantive deviations from the original recommendations.

Analysis of generated responses revealed substantial congruence between LLM-produced recommendations and established clinical guidelines, with 75% (21/28) demonstrating substantial alignment. However, notable divergences were identified: in three instances, the guideline content was significantly more comprehensive than the LLM outputs. Conversely, one LLM-generated recommendation included supplementary clinically relevant insights not present in the established guidelines, while three responses presented direct contradictions to guideline recommendations. In oncology, LLMs and advanced AI demonstrate promising potential to augment multidisciplinary decision-making by generating clinically relevant treatment suggestions that align closely with expert recommendations, though human oversight remains essential. 18 The present findings highlight the potential complementary relationship between AI systems and evidence-based guidelines in clinical practice. While guidelines provide a foundational framework for standardized care, integrating dynamic, personalized insights from AI platforms may enhance clinical decision-making processes in oncology. This synergistic approach could ensure clinicians have access to comprehensive and up-to-date information, facilitating adaptive and evidence-informed treatment strategies tailored to individual patient presentations. Consequently, combining established guidelines with AI-derived insights may significantly optimize therapeutic outcomes in cancer management.

Recommendation 12 considers SND an acceptable alternative in patients with stage T1 or T2 and cN1 oral cavity SCC, and for very selected cN2 cases with positive lymph nodes at levels I or II. However, radical neck dissection (RND) and modified radical neck dissection (MRND) remain the standard of surgical care for patients with advanced T-stage disease or cN2 and cN3 disease. The guideline presents SND as an “acceptable alternative” to CND/RND for early stages, while Claude more definitively “supports” SND as the primary approach. Perplexity specifies levels I-III for SND and suggests possible extension to level IV, while the guidelines don't specify the exact levels to be dissected. Most importantly, Perplexity's response is incomplete as it only recommends levels I-IV for advanced disease, while standard RND should include level V as well, which is correctly implied in the guideline's recommendation.

Recommendation 13 strongly recommends adjuvant concurrent chemoradiation over adjuvant radiation therapy alone primarily for patients with positive surgical margins, metastatic lymph nodes with ENE and the presence of more than two positive lymph nodes (level of evidence 4, grade of recommendation B). Perineural invasion (PNI), vascular embolization, multiple lymph node involvement, and lymph node involvement at low cervical levels (IV and V) could, in selected cases and after multidisciplinary discussion, be considered for concurrent chemoradiation. The presence of multiple involved lymph nodes suggests a more aggressive tumor biology according to Gemini, whereas it is not considered in ChatGPT and Claude responses. Perplexity recommends chemo-radiotherapy in case of N2 or N3 nodal disease. Additionally, the LLM response omits more nuanced recommendations by specifying secondary considerations that could warrant chemoradiation after multidisciplinary discussion. The guideline provides additional considerations for chemoradiation in selected cases after multidisciplinary discussion, and like ChatGPT, it excludes critical scenarios outlined in the guideline. Some factors are not mentioned in recommendation 13 and in the LLM, like higher lymph node density, that correlates with significantly poorer survival and can help identify patients who may benefit from intensified adjuvant therapy after surgery. 19

In contrast, recommendation 10 states that sentinel lymph node biopsy (SLNB) should be performed in patients with stages I and II oral cancer when all the resources, including appropriate preoperative imaging, specialized head and neck cancer radiologist, nuclear medicine services, radiolabeled markers, pathologists, and immunohistochemistry techniques, are available. Otherwise, selective neck dissection should continue to be the standard of care for those patients and active surveillance of the neck is not recommended. The Claude LLM explicitly recommends SLNB for tumors with thickness greater than 4 mm and allows active surveillance in select cases (very early tumors less than 4mm depth or surgically unfit patients). Notably there is a direct contradiction in the responses regarding active surveillance, as Gemini LLM suggests it as a viable option just for “low-risk tumors”.

According to Recommendation 16, there is insufficient evidence to prescribe cetuximab concurrent with radiation therapy in patients with oral cavity cancer who are unsuitable to receive cisplatin. However, both ChatGPT and Perplexity suggest that scientific evidence supports this approach.

It is essential to emphasize that the specificity of responses elicited from LLMs depends on the precise formulation of inquiries. Consequently, the methodological section delineates the prompts utilized in this investigation to ensure procedural transparency and reproducibility. In Oncology decision-making, carefully designed prompts significantly improve LLM output relevance and accuracy, ensuring clinically meaningful recommendations while minimizing errors and enhancing utility in complex therapeutic scenarios. 20

A comparative analysis of guideline development versus AI utilization reveals distinct temporal and methodological disparities. Guideline formulation requires substantial time investment and requires collaborative efforts from multiple domain specialists conducting comprehensive analysis of the contemporary literature on specific clinical topics. Upon publication, guidelines risk becoming outdated as new evidence emerges remaining so until subsequent revisions. In contrast, AI systems provide instantaneous responses, potentially remaining up to date with evolving evidence, depending on the platform's knowledge base update frequency.

AI has considerable potential to enhance multiple dimensions of oral cancer management through incremental integration into clinical practice. The formulation of clinical guidelines is resource-intensive and financially demanding, with revision cycles typically occurring at multi-year intervals During these intervals, substantial scientific advancements may occur. LLMs offer the distinct advantage of facilitating instantaneous consultation capabilities, thus providing access to current evidence-based information.

LLMs should be viewed as complementary tools rather than comprehensive substitutes for established medical guidelines. Despite the 75% concordance in responses, significant limitations persist: Medical guidelines undergo rigorous peer review, systematic evidence evaluation, and expert consensus deliberations that exceed current capabilities of LLMs. LLMs may disseminate outdated or erroneous information due to static training datasets and inability to incorporate new evidence without extensive retraining. Furthermore, medical guidelines incorporate explicit evidence classification systems, recommendation strength, and transparent reasoning frameworks supporting each clinical recommendation – elements that LLMs cannot consistently replicate.

Can LLMs function as complementary tools or potential substitutes for clinical guidelines? The concept involves physicians utilizing point-of-care LLMs to rapidly access information traditionally found in guidelines. To achieve optimal results, clinicians would need to construct customized prompts with appropriate context and specific queries, subsequently refining the outputs to eliminate superfluous information. Nevertheless, the results obtained through this methodology demonstrate clinical utility. The responses generated reflect current recommendations contemporaneous with the query formulation. Conversely, clinical guidelines undergo revision at variable intervals, and medicine inherently operates within a framework of evolving scientific understanding. This transience of medical knowledge is particularly pronounced in the current era, characterized by an unprecedented rate of scientific advancement. Consequently, established guidelines may not consistently reflect the most current evidence base.

This investigation encompasses methodological limitations that warrant acknowledgment. First, the analytical scope was confined to a limited subset of recommendations derived from the guidelines studied. Additionally, the comparative assessment between guideline content and LLM-generated responses may have introduced evaluative bias due to subjective interpretations among experts determining the clinical significance of discrepancies between these information sources.

## Conclusion

The findings of this study indicate that, with optimized query formulation, LLMs achieved 75% concordance with established guideline recommendations. This level of alignment suggests a promising capacity for LLMs to support clinical decision-making in practice. However, comprehensive validation studies are essential to rigorously authenticate the accuracy and reliability of LLM-generated responses before they can be fully integrated into clinical use. Therefore, at this stage, it is prudent to regard these AI systems as valuable complementary tools rather than substitutes for established clinical guidelines.
